# A Custom-Made Surgical Guide for Accurate Enucleation of Nasopalatine Duct Cysts: A Technical Note and Case Report

**DOI:** 10.1155/2023/9246701

**Published:** 2023-08-01

**Authors:** Masako Fujioka-Kobayashi, Naoki Miyasaka, Ayako Miyasaka, Masateru Koyanagi, Ryo Inada, Takahiro Miyasaka, Takafumi Satomi

**Affiliations:** Department of Oral and Maxillofacial Surgery, School of Life Dentistry at Tokyo, The Nippon Dental University, Tokyo, Japan

## Abstract

Nasopalatine cysts are common nonodontogenic cysts that occur in the maxilla. During the nucleation of large cysts extending to the floor of the nasal cavity, care must be taken to avoid damage to the nasal mucosa. In the present report, an innovative custom-made surgical guide made by a Three-dimensional printer is introduced for accurate enucleation surgery. The patient's cone-beam computerized tomography and dental model scan data were obtained, and a tooth-supported type of surgical guide was designed containing a circular plate structure showing the size of the cystic region, an indicator that showed the position of the bottom of the cyst, and a sliding stopper that was used to accurately indicate the position of the deepest cyst wall. The surgical tool enabled us to indicate the accurate size, location of the cysts, and approach direction. Although effective and accurate navigation systems have become increasingly available, the cost-effective and accurate computer-aided design/computer-aided manufacturing surgical guide system introduced in the present report could support the safe enucleation of large nasopalatine duct cysts.

## 1. Introduction

Nasopalatine duct cysts are the most common nonodontogenic cysts in the maxilla [1]. These cysts originate from epithelial remnants in the nasopalatine duct [2]. The etiopathogenesis of nasopalatine duct cysts remains uncertain; however, some reports suggested that they arise due to the spontaneous proliferation of residual epithelial tissue due to several factors, such as mechanical trauma, bacterial infection, or mucous retention [3, 4]. Cyst enucleation is a general treatment for nasopalatine cysts. However, persistent fistulas should be carefully avoided when treating large nasopalatine duct cysts because of the proximity to both the oral and nasal cavities [2].

Three-dimensional (3D) technologies have had an enormous impact on clinical outcomes and on the way clinicians approach treatment planning [5]. Patient volumetric data obtained after medical imaging are used for virtual surgical planning (VSP) and/or patient-specific implant (PSI) design. VSP and PSI have effectively been applied for oral and maxillofacial surgeries, including mandibular reconstruction, orthognathic surgeries, orbital floor fracture surgeries, and dental implant treatments [5, 6]. For instance, 3D-printed surgical guide tools were previously reported for the surgical treatment of a compound odontoma complicating the adjacent tooth structures or ossifying fibroma closely positioned to the inferior alveolar nerve [7, 8]. However, those 3D-printed surgical guides indicate the accurate approach position on the bone surface but not the depth of the lesion for surgical treatments.

In the present report, we introduce a custom-made 3D-printed surgical guide that not only shows the location of the cyst but also navigates to the deepest position, an important point of the cyst enucleation procedure.

## 2. Case Presentation

**Figure 1:**
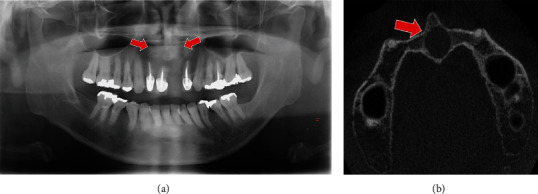
A well-defined, unilocular radiolucent lesion in the maxillary anterior region on (a) the panoramic radiograph and (b) CBCT. The red arrows indicate the cystic lesions on the X-ray images.

A 56-year-old man was referred by the dentist to the Department of Oral and Maxillofacial Surgery, Nippon Dental University Hospital, concerning a nasopalatine duct cyst. He did not have any complaints or relevant medical history. The panoramic radiograph and cone-beam computed tomography (CBCT) showed a well-defined unilocular radiolucent area beyond the nasal floor (Figure 1). Based on an assessment of the location, presenting symptoms, and radiographic findings, the clinical diagnosis indicated a nasopalatine duct cyst. Nonetheless, the differential diagnosis encompassed odontogenic tumors, periapical cysts, granulomas, odontogenic cysts, and nasolabial cysts. Cystectomy was chosen for the treatment, and the tooth-supported one-piece surgical tool was designed to avoid damaging the nasal mucosa.

**Figure 2:**
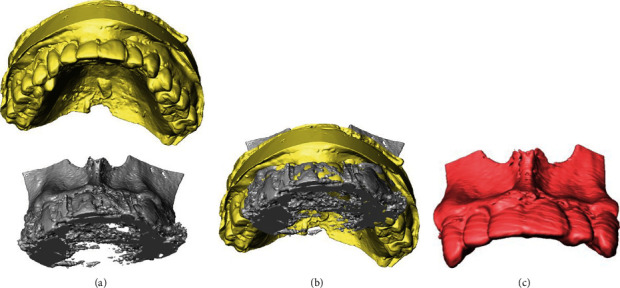
Chart of the segmentation procedure of the maxilla. (a) STL file of the scanned maxillary dental model and 3D-reconstructed CBCT volume data of maxillary bone were (b) laid to overlap each other, and (c) merged on the Amira® software.

Transferred digital imaging and communication in medicine data of the patients' CBCT and stereolithography (STL) files of dental model scans were obtained preoperatively. The volumetric data of the maxilla and dentition model data were segmented and merged, and a surgical guide was designed by 3D structural analysis software (Amira®, Thermo Fisher Scientific, Waltham, MA, USA; Figure 2).

**Figure 3:**
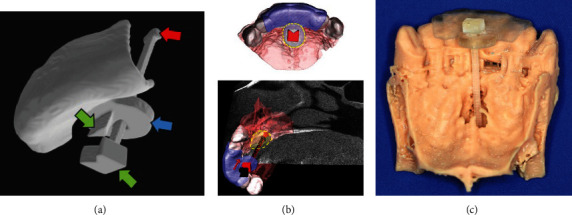
The design of surgical guide for nasopalatine cyst removal. (a) The tooth-supported one-piece surgical guide includes (1) the circle structure (blue arrow), (2) the stopper (green arrows), and (3) the nasal side of the indicator (red arrow). (b) The circle structure in the plate part indicates the maximal cyst size when observed from the occlusal plane. The indicator can slide and show the direction toward the bottom of the cyst. (c) The clear-colored surgical guide printed by the 3D printer was checked for insertion into the bone model of the maxilla (brown).

A tooth support structure was designed without undercuts, similar to a mouthpiece (Figure 3). A circular plate structure was further made to fit the size of the cystic lesion when observed from the occlusal plane as the surgeon approached. An indicator structure that shows the position of the bottom of the cyst was then designed with two stoppers on both sides—the nasal and tooth sides. The stopper on the tooth side of the circular plate was set to allow the dull tip on the nasal side to stop at the position of the deepest cyst wall accurately. The slot design was provided for the circle plate, as the indicator bar could slide in the circle plate along the approach direction. Furthermore, the indicator with a triangular prism shape was designed not to turn around in the slot when sliding.

The designed surgical guide was exported as an STL format file and printed by a tabletop 3D printer (Form 3B, Formlabs, Somerville, MA, USA) using biocompatible surgical guide resin (Form 3B), followed by posttreatment with rinsing in 99% isopropyl alcohol and postcuring for 20 minutes according to the manufacturer's protocols (Figure 3(c)).

**Figure 4:**
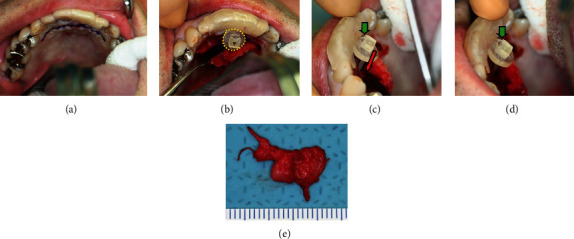
The surgical procedure using the surgical guide. (a) The incision line was shown. (b) Intraoperative image showing the size of the cyst with the circle structure of the surgical guide. (c) The direction and depth of the cyst could be checked with the surgical guide. The indicator can slide the direction showing as the red arrow. (c and d) The stopper (green arrows) confirmed if the bottom part of the cyst was accurately removed. (e) The resected specimen.

The custom-made surgical guide was placed and used (1) before and (2) after mucoperiosteal dissection and exposure of the palatine bone and (3) after cystectomy (Figures 4(a), 4(b), 4(c), and 4(d)). The cyst was carefully removed, and the specimen was sent for pathological microscopic evaluation (Figure 4(e)). During the procedure, the nasal mucosa was exposed after cyst resection; however, unusual bleeding from the nasal mucosa was not observed. The wound was thoroughly rinsed and closed primarily with resorbable Vicryl® sutures. The patient did not experience postoperative bleeding or other complaints. Pathological investigation revealed a nasopalatine duct cyst. A stable clinical status was observed postoperatively.

## 3. Discussion

Recently, virtual reality (VR), augmented reality (AR), and mixed reality systems in surgical simulation have become increasingly available for effective and accurate surgeries [9]. AR in maxillofacial surgery is beneficial for preoperative planning to provide practical outcome forecasts and intraoperative navigation to minimize possible risks [10]. AR has been extensively developed in dental implantology [9, 10]. Lin et al. reported that the stereoscopic visualization concept combined with head-mounted displays successfully increased the accuracy of computer-aided implant surgery [11]. A pilot-clinical analysis by Pellegrino et al. further demonstrated the possibility of the use of a virtual display for dynamic navigation during dental implant surgery [12]. In the field of orthognathic surgery, the stereo camera-based AR navigation system was applied for accurate Le-Fort I osteotomy during surgery or diagnosis [13]. However, such systems complicate registration preparation, are expensive and time-consuming, and more importantly, in some applications, are not yet very reliable for accuracy without cross-checking by conventional surgical methods [13].

The main indications of 3D-printed surgical guide tools in oral and maxillofacial surgeries include dental implant surgery, mandibular reconstruction, orthognathic surgery midface reconstruction, Temporomandibular Joint reconstruction, maxillofacial prosthodontics, and patient communication or training and clinical education [5, 14]. In dental implant surgery, the most printed 3D tools are surgical guides designed to facilitate the orientation and execution of drillings, permitting correct implant placement, as predicted preoperatively [14]. Furthermore, the printed anatomic models are useful to practice the procedure preoperatively and to preshape the osteosynthesis or reconstruction plates in orthognathic surgeries, tumor resection surgeries, and reconstructive surgery in the jaws.

The use of surgical guides for cysts or benign tumors has previously been reported, and two types, drilling location guides and osteotomy locating guides or cutting guides have been introduced [7, 15–17]. One of the biggest benefits was a more precise localization of the bone lesion and a better precision of the osteotomy tracing, resulting in a less invasive approach [18]. Furthermore, the cutting guide allows the surgeon to reposition the bone lid after lesion removal, thus enabling a bone-sparing approach [18]. In the present report, 3D-printed surgical guides displayed not only the accurate approach position on the bone surface but also the depth of the lesion, which allowed safe enucleation of large nasopalatine duct cysts. The tooth-supported surgical guide was selected for the design since the tooth-supported guides are considered to have the highest accuracy compared with the bone-supported or mucosa-supported surgical guides [19]. Furthermore, the designed surgical guide in the present report has a one-piece structure, but the indicator bar could slide along the direction of the lesion and stop on the bottom border of the cyst. This type of surgical guide is introduced in the field of oral and maxillofacial surgery.

The limitations of 3D-printed surgical tools include the fact that preoperative planning takes more time, is more complex, and requires a certain degree of knowledge and techniques in computer sciences. However, 3D-printed anatomic models and surgical guides surely reduce operative time. It was reported by Ballard et al. using literature-based financial analyses that medical 3D printing appears to reduce operating room costs secondary to shortening procedure times [20]. Furthermore, it should be noted that surgical guides are considered invasive medical devices due to their penetration into the body through the bone, mucosa, or skin during the operation, even for temporary or short-term use [14].

The assumed benefit of computer-assisted surgical planning and subsequent preparation and use of the surgical guide tool must be a thorough preoperative diagnosis and a more predictable procedure concerning anatomical structures. The introduced 3D-printed surgical guide system indicating the direction of the surgical approach and the depth of the lesion might be able to replace real-time navigation systems without requiring expensive tools or additional time for registration of the patient's preoperative CT image dataset in certain cases.

## 4. Conclusions

The 3D-designed custom-made surgical guide could help surgeons easily approach cysts and confirm the deepest position of the cyst walls to avoid injuring the nasal mucosa via an oral approach. This kind of surgical tool to determine the depth could be applied to various cases, including cysts or tumors, in the field of oral and maxillofacial surgery in the future.

## Data Availability

Data supporting this research article are available from the corresponding author on reasonable request. The data are not publicly available due to the ethical issue.
